# Plant impedance spectroscopy: a review of modeling approaches and applications

**DOI:** 10.3389/fpls.2023.1187573

**Published:** 2023-07-31

**Authors:** Maxime Van Haeverbeke, Bernard De Baets, Michiel Stock

**Affiliations:** Knowledge-Based Systems (KERMIT), Department of Data Analysis and Mathematical Modelling, Faculty of Bioscience Engineering, Ghent University, Ghent, Belgium

**Keywords:** electrochemical impedance spectroscopy, plant cells, equivalent electrical circuit, plant stress, fruit ripening

## Abstract

Electrochemical impedance spectroscopy has emerged over the past decade as an efficient, non-destructive method to investigate various (eco-)physiological and morphological properties of plants. This work reviews the state-of-the-art of impedance spectra modeling for plant applications. In addition to covering the traditional, widely-used representations of electrochemical impedance spectra, we also consider the more recent machine-learning-based approaches.

## 1 Introduction

Electrochemical impedance spectroscopy (EIS) is a popular method to characterize the electrical properties of (bio-)electrochemical systems. Applying an alternating voltage input^1^ V(f) to a plant tissue sample gives rise to the flow of an electric current I(f) through the cell walls, between cells, and in the plant fluids. The polarization processes caused by resistive and capacitive elements in the plant tissue impede the flow of this electric current, resulting in a phase shift between the current and voltage phasors. This phenomenon is the electrochemical impedance of the sample and can be measured at multiple frequencies f using an analyzer. The impedance Z(ω) , where ω=2πf , is a complex-valued quantity that relates to the current and voltage as


(1) $$Z\left(\omega \right)=\frac{V\left(\omega \right)}{I\left(\omega \right)}=\left|Z\left(\omega \right)\right|\left(\cos(\phi \left(\omega \right)\right)+j\sin(\phi \left(\omega \right)))\;,$$

where |Z(ω)| is the magnitude of the impedance and ϕ(ω) is the phase angle between the current and the voltage. There is ample evidence that the measurement of Z(ω) over a range of frequencies (giving rise to an impedance spectrum) holds promise in ascertaining a range of plant physiological properties (Jócsák et al., 2019), including various types of plant stress (Lichtenthaler, 1998). As ω=2πf , a function in terms of ω is also a function of f . In the remainder of this work, we express the impedance as a function of f to make the dependence on the input frequency in Hz explicit. The non-invasive nature of impedance spectroscopy measurements allows for the influence of an (a)biotic stressor to be analyzed without being confounded by damage caused by the measurement technique.

Phospholipid bilayer membranes in plant cells act as capacitors, forming an electrical double layer at their interfaces when exposed to an electrical field. Charge-carrying molecules in the symplast (i.e., the intracellular matrix) and apoplast (i.e., the extracellular matrix) electrolytes give rise to electrical resistances. When an alternating voltage input pulse is applied, the electrochemical components in the plant cells undergo polarization and subsequent dielectric relaxation. The nature and intensity of this polarization depend on the properties of the measured tissue and the interrogated frequency. At low frequencies, the capacitive membranes obstruct current flow into the cells, and the resulting current only flows through the extracellular matrix. At higher frequencies, the current can penetrate the cells, allowing for the interrogation of the intracellular impedance caused by polarization processes in the cytoplasm, which contains charged molecules and capacitive membrane structures. Several published works provide visualizations of this phenomenon (Azzarello et al., 2006; Ehosioke et al., 2020; Liu et al., 2021a; Cheng et al., 2022). Changes in plant physiology are reflected in the different polarization processes and their corresponding relaxation. All this information is contained within an adequately measured electrochemical impedance spectrum. The challenge addressed by plant EIS modeling is to extract this information from the impedance spectra to attain a valuable characterization of the considered plant system.

Two types of plot are typically used to visualize impedance spectra: *Nyquist plots*, which display the real values of the impedance measurements as a function of their imaginary components, and *Bode plots*, which display the magnitude and the phase of the impedance as a function of the measured frequencies (Orazem and Tribollet, 2008; Barsoukov and Macdonald, 2018; Wang et al., 2021). Cole–Cole plots are also used to represent impedance spectra and originate from a 1941 publication by the Cole brothers (Cole and Cole, 1941). They display a material’s complex dielectric constant (related to its impedance) over the measured frequencies. Its real and imaginary parts are represented on the x - and y -axis, respectively. Cole–Cole plots are often confused with Nyquist plots, as they are both Argand diagrams. One drawback of Nyquist and Cole–Cole plots is that they do not provide explicit frequency information. This is sometimes mitigated by adding an indication of the frequency to the observations in the plot. This is done by placing the frequency values next to the observations in the plots. Alternatively, a 3D representation is used, where the frequencies are included as an additional axis (Macdonald et al., 1981; Arteaga et al., 2021).

Given the enormous waste of agricultural produce in the destructive assessment of in-field and post-harvest product quality due to the requirement of a sufficiently large sample size, there is an ever-growing need for clean and effective non-invasive product evaluation methods (Vanoli and Buccheri, 2012; Lakshmi et al., 2017). A growing body of research promotes the use of EIS-based product assessment in this regard (El Khaled et al., 2017; Ibba et al., 2020). While there is expensive commercial multi-modal equipment for EIS, effective custom setups within a price range of a few hundred USD have been used successfully in agricultural applications (Basak et al., 2020a). In parallel with developments in sensors and equipment, the correct and in-depth modeling of plant impedance spectra will allow for the unravelling of the unexplored potential of EIS further.

A panoply of plant phenotyping methods has been developed to investigate various properties related to the agricultural yield and health status of crops. Each of these methods has benefits and drawbacks. An often-reported comparative advantage of EIS in plant characterization studies is that it is less sensitive to environmental influences (Hussain et al., 2021). **Table 1** presents a non-exhaustive overview of plant phenotyping methods for which EIS holds promise as a complementary or competitive analysis method. This table does not include Electrical Impedance Tomography (EIT), Spectral Induced Polarization (SIP), or single-frequency impedance measurements, which we consider to be specific variants of EIS rather than complementary or competitive methods. We refer to other works for more in-depth discussions on non-destructive plant phenotyping methods (Zerbini, 2006; Rahaman et al., 2015; Ali et al., 2017; Lakshmi et al., 2017; Ali et al., 2019).

**Table 1:** Plant characterization methods for which EIS holds promise as a competitive or complementary method.

Category	Method	Application(s)	Strengths	Weaknesses
Molecular	(RT-)(q)PCR	disease (Korimbocus et al., 2002)	reliable	invasive, laborious
	DNA hybridization	disease	reliable	invasive, laborious
Serological	ELISA	disease	reliable	expensive, invasive, complex, laborious
Chemical	Kjeldahl digestion	nutrition	reliable (Muñoz-Huerta et al., 2013)	invasive, slow, *ex situ*
	Dumas combustion	nutrition	reliable (Muñoz-Huerta et al., 2013)	invasive, slow, *ex situ*
	soluble solid content	sugar content and fruit maturity	reliable	invasive, *ex situ*
Optical	VIS/IR spectroscopy/imaging	water content (Zhang et al., 2012; Jin et al., 2017), nutrition, disease (Sankaran et al., 2011)	non-invasive	environment-sensitive
	quantum cascade lasers	disease (Mur et al., 2011)	reliable, quantitative	complex, expensive, *ex situ*
	FT-IR spectroscopy	disease (Abramovic et al., 2007; Liaghat et al., 2014), abiotic stress (Yu et al., 2018), nutrition (Butler et al., 2017)	*in situ* (Abramovic et al., 2007)	environment-sensitive, expensive
	thermal imaging	disease (Chaerle et al., 2006; Xu et al., 2006), drought stress (Hashimoto et al., 1984; Cseri et al., 2013), nutrition (Chaerle et al., 2007)	non-invasive, large-scale	environment-sensitive (Humplík et al., 2015)
	soil-plant analyses development	nutrition (Yue et al., 2020)	fast, non-invasive	limited applicability, environment-sensitive
	hyper-/multispectral imaging	water content (Kovar et al., 2019), disease (Schubert et al., 2001), fruit maturity (Gutiérrez et al., 2019), osmotic stress (El-Hendawy et al., 2019; Mishra et al., 2019; Pieters et al., 2020)	non-destructive, broadly applicable	complex, expensive
	Quickbird satellite	nutrition (Wu et al., 2007; Bausch et al., 2008), disease (Jacobi et al., 2005)	large scale	environment-sensitive, expensive
	colorimetry	nutrition (Hytönen and Wall, 2006; Sahrawat et al., 2016), ripeness (Gonçalves et al., 2007), sugar content (Buysse and Merckx, 1993), stress (Bacci et al., 1998)	fast, cheap	environment-sensitive, invasive
	digital image analysis	disease (Mohanty et al., 2016; Tm et al., 2018; Singh et al., 2019; Liu and Wang, 2021; Nirmal et al., 2022), fruit quality (Benalia et al., 2016), nutrition (Chen et al., 2019)	non-invasive	environment-sensitive
	fluorescence spectroscopy/imaging	disease (Lins et al., 2009; Bürling et al., 2012; Granum et al., 2015; Pérez-Bueno et al., 2015; Montero et al., 2016), mechanical stress (Belasque et al., 2008; Pérez-Bueno et al., 2019), nutrition (Agati et al., 2015)	non-invasive	non-robust (Li et al., 2014)
Electrical	ion-selective sensors	nutrition	fast	invasive
	electronic nose	fruit maturity (Di Natale et al., 2001; Li et al., 2009), disease (de Lacy Costello et al., 2000)	portable, real-time, fast, non-invasive	non-robust, environment-sensitive
	conductivity	nutrition (Bodale et al., 2021)	cheap	invasive, qualitative
	resistance measurements	abiotic stress (Mancuso, 2000)	quick, easy and non-destructive	non-robust
	capacitance measurements	water uptake (Afzal et al., 2017; Cseresnyés et al., 2022), biomass (Dietrich et al., 2013; Cseresnyés et al., 2018)	*in situ*, fast, non-invasive	environment-sensitive
	resistivity tomography	biomass (Paglis, 2013), water content	non-invasive, broad applicability, *in situ*	complex, expensive
Sonic	sonic tomography	disease (Ishaq et al., 2014)	in-field, non-invasive, reliable	complex
Physical	pressure bomb	water potential	reliable	invasive
	penetrometry	fruit maturity	quantitative	invasive

An overall impedance spectrum is obtained when conducting impedance measurements over a range of frequencies. Yet, apart from the electrochemical response of the system under investigation, it can often also include other factors that affect the impedance, such as artefacts and influences due to the experimental equipment. When conducting EIS measurements, the experimental setup must be carefully considered, as it will significantly impact the appropriate choice and performance of the subsequent modeling and analysis. Important considerations include the electrode configuration, the applied frequency range and resolution, the use of minimally interfering connecting cables, and the environmental conditions. Appropriate measures should be taken to address the challenge of decoupling the measurement equipment from the system under test, as well as to ensure that the linearity, stability, and causality requirements for EIS measurements are satisfied. We refer to several excellent recent reviews for in-depth discussions covering the above-mentioned experimental considerations (El Khaled et al., 2017; Prasad and Roy, 2020; Wang et al., 2022; Lazanas and Prodromidis, 2023). In this review, we closely examine the contemporary modeling approaches for EIS in plant applications and aim to provide direction for the recent emergence of machine learning applications in the field.

The paper’s scope covers modeling approaches and data analysis techniques for plant impedance spectroscopy and their application areas. The remainder of this paper is organized as follows. Section 2 provides an overview of plant EIS applications, organized according to the measured organ of the plant. An extensive survey of equivalent electrical circuit modeling approaches for plant characterization and their interpretation is given in Section 3. Section 4 constitutes a thorough review of statistical and predictive modeling methods used for the impedimetric analysis of plants using EIS. Section 5 contains a critical discussion of the preceding sections. The conclusions of this work and suggested future directions of the field finalize this paper in Section 6.

## 2 Overview of plant EIS applications

**Figure 1:**
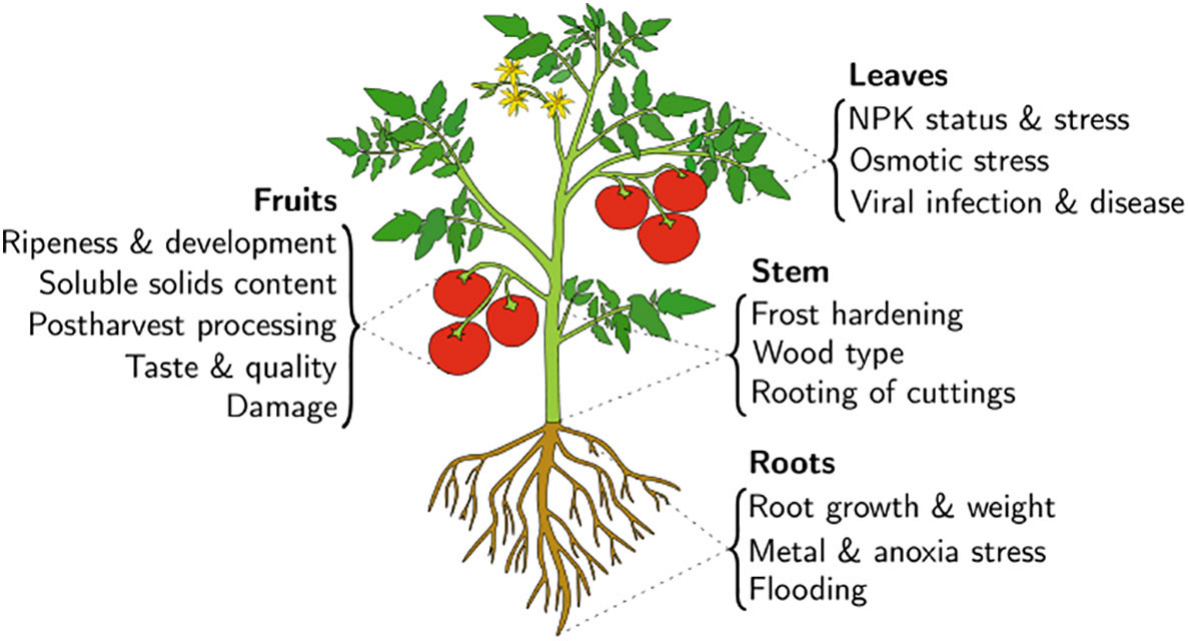
An overview of a number of EIS applications organized according to the organ of the plant at which the electrodes are typically placed for interrogation.

In precision agriculture, the knowledge of external conditions (e.g., soil and air properties) is insufficient to make informed fertilizer and irrigation management decisions (Nowak, 2021). Only interrogation of the plant itself can provide an adequate indication of its physiological state. This statement is the basis for the Speaking Plant Approach (SPA) proposed by Udnik ten Cate in the late 70s (Udink ten Cate et al., 1978). The use of polyvalent EIS-based sensors holds promise for use in precision farming. Here they can enable fertilizer application to specific areas in the farm and inform various farming management decisions when combined with a range of other collected data in a “smart farm” using the Internet of Things (IoT) (Elijah et al., 2018). As a robust, non-destructive, and inexpensive method, EIS provides the means to conduct analyses that conform with the SPA. The applications of EIS to plants are numerous. EIS measurements are typically conducted at the leaves, fruits, stems or roots, depending on the considered application. To our knowledge, no studies report measurements of a plant’s flowers. A general overview of some plant properties and where they can be indirectly measured through EIS is displayed in **Figure 1**. Aside from direct plant measurements, several works explored soil EIS measurements for agricultural applications. These include soil moisture content and indirect plant biomass determination (Wang et al., 2019).

### 2.1 Roots

Roots are the plant organs responsible for interacting with the soil to secure water, minerals, (micro)nutrients, and, for some species, symbiotic nitrogen-fixing bacteria. A comprehensive analysis and understanding of roots are essential to investigate the complex interactions of plants with the soil and the climate. Electrical methods hold promise in overcoming the difficulties in accessing root properties (Ehosioke et al., 2020). An accurate and thorough characterization of plant root systems allows for the establishment of optimal use of water and fertilizer, assuring maximal crop quality and yield. Particularly in light of current global environmental issues such as food waste, inadequate fertilization and irrigation management, there is a need for in-depth, fast, easy, non-destructive, and *in situ* methods to evaluate the morphological and physiological properties of plant roots. The discovery that there is a linear relationship between the capacitance of a plant’s roots and their size in the last century prompted the ever-growing interest in investigating plant roots by measuring their electrochemical properties. Ozier–Lafontaine and Bajazet (2005) established strong correlations between the capacitance of root tissue and the (wet and dry) weight of the root system. The EIS method has demonstrated its potential to indirectly assess and monitor the root biomass, morphological indices (Cao et al., 2011), and environmental stressors, including cadmium pollution, alkali stress, drought, freeze–thaw damage (Repo et al., 2016), cold acclimation (e.g., frost hardening), osmotic stress, root hypoxia (Vozary et al., 2012) (e.g., caused by flooding; Jócsák et al., 2010), root mycorrhizal colonization (Repo et al., 2014), and weed competition. Liu et al. (2021a) recently conducted an in-depth review of progress and developments in applying EIS to plant roots.

### 2.2 Stem

The stem of a plant grants it structural strength while providing a means for the unidirectional upwards transport of water to the leaves and fruits and the bidirectional transport of assimilates, other nutrients, and signaling molecules through the phloem. EIS measurements have been conducted at the stems of plants to investigate a range of phenotypical properties. Direct plant monitoring is one of the strategies in precision agriculture to improve crop yield, working towards food security for the increasing global population. Bar-On et al. (2021) recently developed an EIS method for *in-vivo*, *in situ*, and non-destructive monitoring of a plant’s physiological status. It is based on a four-electrode setup attached to the stem of a *Nicotiana tabacum* plant used as a dicot plant model.

Tomkiewicz and Piskier (2012) proposed a nutrition index, calculated using the impedance magnitude values measures at the stem of tomato plants over a frequency range of 1.5 kHz to 16 kHz. This constitutes an initial step towards site-specific fertilizer management in greenhouses. Borges et al. (2012) provided initial evidence for the potential of EIS for early detection of plant diseases. Differences in impedance spectra (measured at the stem) were observed for young pine plants infected with the nematode *Bursaphelenchus xylophilus* (Borges et al., 2012). The rooting potential of shoot cuttings was investigated by Mancuso (1999) through double-DCE equivalent electrical circuit models. Recently, Astashev et al. (2022) also developed a sensor and model to conduct *in situ* EIS measurements evaluating the physiological condition of trees. Branch length, tree health, and effects of drying and grafting were all impedimetrically evaluated. Aouane et al. (2021) evaluated EIS as a method to monitor evapotranspiration on a celery stalk. They found that the extracellular resistance from a Cole EEC model can describe different stages of water loss and nutrient depletion.

### 2.3 Leaves

Leaves assimilate the plant’s carbohydrates through photosynthesis. They also regulate the flow of water during evapotranspiration. The water potential of plants is an essential parameter to model a range of physiological processes (De Swaef et al., 2022). The non-invasive attachment of electrodes to plant leaves allows for real-time *in-vivo* monitoring of several important plant properties using EIS. EIS has been proposed to evaluate a plant’s water status and potential. Basak et al. (2020a) modelled the relative water content (RWC) of canola, corn, wheat, and soybean leaves using multivariate linear regression with impedance magnitude features at different frequencies that were selected using backward elimination. They conducted a similar analysis for the leaf nitrogen content of these crops (Basak et al., 2020b). To that end, they used a portable and relatively cheap device. Xing et al. (2021) demonstrated a strong correlation between the impedance of leaves and their water status through the cell elasticity, which was measured by leaf clamping with different gripping forces. The authors used polyethylene glycol (PEG) to induce different osmotic stress levels in *Orychophragmus violaceus* in their experiments. Ali Solangi et al. (2021) linked plant capacitance to the vacuole and cell volume in their study of mangrove plants’ salt storage capacity. Recently, Nouaze et al. (2022) exhibited the potential of EIS for real-time *in-vivo* physiological monitoring of lettuce, while Sugiyama and Okajima (2022) demonstrated that the solar illumination of plants is reflected in their impedance spectra.

### 2.4 Fruits

The fruits of a plant contain the seeds required for reproduction. This is of particular commercial interest due to its use as a food product and other commodities. Applications of EIS measurements on fruits include fruit maturity and firmness (Ivanovski et al., 2020) and the composition and cell vitality of fruits (Caravia et al., 2015). Caravia et al. (2015) found that the impedance of Shiraz grapes follows the accumulation of total suspended solids during cell death in berries at a late ripening stage. They also conducted experiments to electrochemically evaluate changes in veraison grapes to 110 days after anthesis and assessed the effects of freezing and thawing on grape impedance spectra. EIS has also been applied to evaluate the effects of processing agricultural products (e.g., drying and freeze–thawing). The freezing of agricultural products causes the formation of ice crystals in the plant tissues. These ice crystals cause cell membrane rupturing, resulting in the loss of the latter’s capacitive properties. The impedance is further decreased upon thawing due to leakage of the intercellular matrix to the extracellular space. As the intracellular medium is less resistive than the extracellular matrix (Lee and Watanabe, 2022), this causes a substantial decrease in the latter after plasmolysis and the resulting electrolyte leakage. Wu et al. (2008) did such an evaluation on eggplant pulp. Applying EIS for fruit status appraisal will allow for further development and automation in horticulture (e.g., automatic picking of ripe fruit with robotic arms; Park et al., 2022).

Watanabe et al. recently proposed a feature extracted from the Nyquist plot to assess damage in biological tissues during the processing of agricultural products (Watanabe et al., 2018):


(2) $$\text{LTO}=\left|Z_{\text{top}}\right|=\sqrt{{\left(Z_{\text{top}}^{\text{Re}}\right)}^{2}+{\left(Z_{\text{top}}^{\text{Im}}\right)}^{2}}\;.$$

This feature is the length of the impedance vector $Z_{\text{top}}$ where the circular arc in the Nyquist plot reaches its zenith and is called the LTO (Length Top Origin). The LTO has been used to impedimetrically assess treatments of mechanical bruising, hydrostatic pressure, and freeze damage of Japanese pears (Watanabe et al., 2018; Lee et al., 2019; Lee and Watanabe, 2022). The authors reported a correlation between the LTO and the electrical resistance value of the extracellular matrix. The fruit tissues analyzed using the LTO only showed a single impedance arc in the Nyquist plot. While a correlation was found with the resistance of the extracellular matrix, the LTO remains somewhat arbitrary. More principled indicators, such as the cell disintegration index presented and derived in Angersbach et al. (1999), should be preferred. This index indicates the degree of cell permeabilization (i.e., disintegration) based on changes in the conductivity behavior of the sample. The cell disintegration index assumes an equivalent electrical circuit model. Such models are discussed in the next section.

## 3 Plant equivalent circuit modeling

Equivalent electrical circuits (EECs) are one of the earliest-developed and most successful tools for analyzing plant EIS measurements. A myriad of equivalent electrical circuits for analyzing biological tissues have been proposed since the second middle of the last century. They are currently still the most widely used models in EIS analysis, albeit they are subject to some criticism. One such criticism is that there is degeneracy in EECs: multiple EEC configurations are capable of modeling a given set of EIS measurements. Several examples of such “degenerate equivalent circuits” were compiled by Fletcher (1994). Apart from achieving a high-quality fit, care must be taken to use circuit models with a clear biophysical meaning without being more elaborate than they should be. An appropriate EEC can provide insights into a variety of plant physiological processes. Furthermore, when fit to the impedance measurements, the parameters of an EEC are effective at summarizing the information present, making them valuable features in statistical models and diagnostic tools. As such, the physiological state of a plant system can be monitored through the tracking of EEC parameters.

### 3.1 Plant equivalent circuit configurations

The three circuit components typically encountered in a plant EEC are resistors (R), capacitors (C), and constant phase elements (CPE). Their respective impedance expressions are given by:


(3) $$Z_{\text{R}}\left(f\right)=R$$

(4) $$Z_{\text{C}}\left(f\right)=-j\cdot \frac{1}{2\pi fC}$$

(5) $$Z_{\text{CPE}}\left(f\right)=\frac{1}{Q\left[\cos(\frac{\pi }{2}\alpha \right)+j\sin(\frac{\pi }{2}\alpha )]{\left(2\pi f\right)}^{\alpha }}=\frac{1}{Q{\left(2\pi jf\right)}^{\alpha }}.$$

Here, f is the frequency, R is the resistance, C is the capacitance, and Q and α∈[0,1] (dispersion or distribution coefficient, a measure of deviation from ideal capacitive behavior) are the two parameters associated with the CPE. CPEs get their name from their property of giving rise to impedance measurements whose phase angle is independent of the frequency but dependent on the parameter α . The Warburg element is a special case of a CPE, where α=0.5 . It is commonly used for modeling diffusion processes in mass transfer. The impedance expressions in Eqs. (3)–(5) are used together with Kirchoff’s laws to obtain the impedance expressions of the EEC.

**Table 2** displays the *simple Voigt circuit* and the single and double shell models (derivations of) that are applied in most plant EIS applications. Voigt circuits are often serially expanded to contain additional parallelly connected resistors and capacitors so as to model different parts of the considered plant organ. The substitution of capacitors with CPEs in practical applications is a trend in the field of electrochemical power sources that has recently also been picked up in plant applications. Equivalent electrical circuits containing CPEs are commonly called fractional-order circuit models (Freeborn, 2013), whereas EECs consisting of resistors and capacitors are integer-order circuit models. In the literature, these two categories of EECs are often called lumped and distributed models, respectively. The Voigt circuit is often fractionalized to model complex bioelectrochemical processes by substituting the capacitor with a CPE, forming the *single dispersion Cole model*. The single dispersion Cole model is often expanded in series with another parallelly connected resistor-CPE element, resulting in the *double dispersion Cole model*. The impedance expression of the Cole models is given by

**Table 2:** The basic equivalent electrical circuit models (derivations and modifications of) which are commonly used in the modeling of plant EIS measurements.

Equivalent electrical circuit	Description
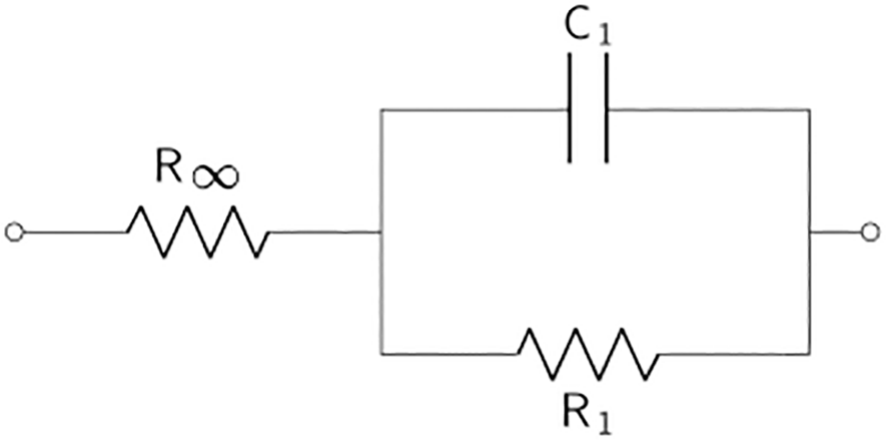	The simple *Voigt* or Debye circuit without inductor. The *single dispersion Cole model* proposed by Cole (1940) can be seen as the fractional elaboration of Voigt circuits, where a CPE replaces the capacitor. This is one of the oldest fractional circuit models and is often used to model biosystem impedance spectra. In that case, *R*_∞_ is the high-frequency resistance, and *R*_1_ + *R*_∞_ is the low-frequency resistance. A drawback is the limited biological interpretability.
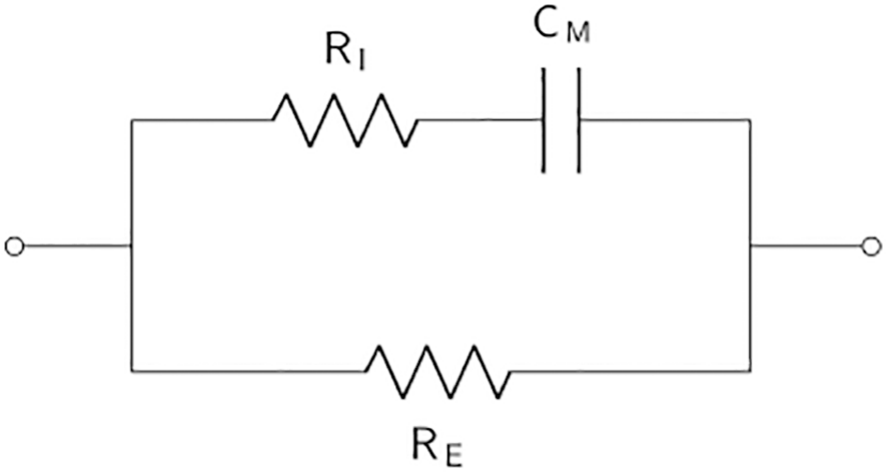	The *single shell model* (Toyoda and Tsenkova, 1998), also called the *simplified Hayden model* (Hayden et al., 1969). *R*_E_ is the extracellular resistance of the apoplastic fluid, *R*_I_ is the intracellular resistance, and *C*_M_ is the capacitance of the cell membrane. The name of the model was coined by Zhang et al., contrasting it with their proposed double shell model (Zhang and Willison, 1992).
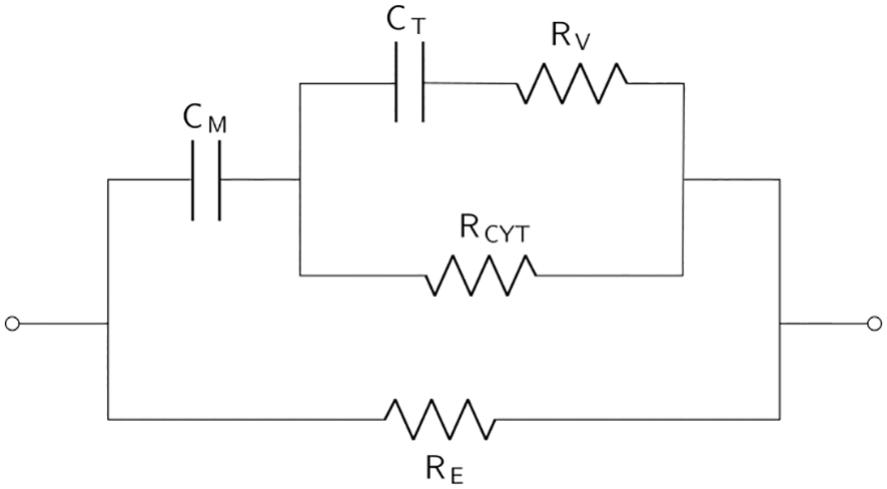	The *double shell model* proposed by Zhang and Wilson (1991). *C*_M_ is capacitance of the cell membrane, *C*_T_ is the capacitance of the tonoplast, *R*_CYT_ is the cytoplasmic resistance, *R*_V_ is the vacuolar resistance, and *R*_E_ is the extracellular resistance. In some works, *R*_V_ is referred to as the resistance of the cell wall (Harker and Maindonald, 1994).


(6) $$Z_{{\text{Cole}}_{\text{N}}}\left(f\right)=R_{\infty }+\sum_{n=1}^{N}\frac{R_{n}}{1+{\left(2\pi jf\right)}^{\alpha }R_{n}Q_{n}}$$

where N is the number of resistor-CPE element elements included in the model. N=1 for the single dispersion Cole model and N=2 for the double dispersion Cole model. The Cole models are simple and often fit the measurements well due to their fractional elements. They have been applied in various plant EIS applications (Wu et al., 2008; Mousa et al., 2019; Aouane et al., 2021).

The ZARC element (Macdonald, 1992), also referred to as the Distributed Circuit Element (DCE), is an alternative element often used in EECs. Its impedance expression $Z_{\text{ZARC}}\left(f\right)$ is similar to that of the resistor-CPE components discussed above, but is expressed in terms of time constants rather than capacitances:


(7) $$Z_{\text{ZARC}}\left(f\right)=\frac{R_{0}}{1+{\left(2\pi jf\tau \right)}^{\alpha }}\;,$$

where τ is the mean time constant. Note that the resistor-CPE element in Eq. (6) and the ZARC element in Eq. (7) coincide when $R_{n}Q=\tau^{\alpha }$ . As such, an alternative formulation of the double (or higher order) Cole model is a resistor in series with two (or more) ZARC elements. Such models, where each ZARC element models a specific part of a plant’s organ, are often applied in plant EIS research. Some examples are the papers by Repo et al. (1994; 2002), Ozier–Lafontaine and Bajazet (2005), Vozary et al. (2007; Vozáry and Benko, 2010), and many more (Mancuso and Rinaldelli, 1996; Mancuso, 1999).

The *single shell model* (see **Table 2**) is a simple EEC of plant cells, considering the cell membrane’s capacitance and the extra- and intracellular resistance. Its impedance expression is given by


(8) $$Z_{\text{single}\;\text{shell}}\left(f\right)=\frac{R_{\text{E}}\left(1+2\pi jfC_{\text{M}}R_{\text{I}}\right)}{1+2\pi jfC_{\text{M}}\left(R_{\text{E}}+R_{\text{I}}\right)}\;,$$

where $R_{\text{E}}$ is the extracellular resistance, $C_{\text{M}}$ is the capacitance of the cell membrane, and $R_{\text{I}}$ is the intracellular resistance.

There are two commonly applied modifications of the single shell model. The first one is the Hayden model, where an additional resistor is parallelly connected to the cell membrane capacitance $C_{\text{M}}$ (Hayden et al., 1969). As the Hayden model predates the single shell model, the latter is sometimes called the simplified Hayden model. The simplification of the Hayden model by the omission of the additional resistance is motivated by the observation that this resistance is usually much larger than the other two resistances, as shown by Cole (1968). The second modification of the single shell model is the replacement of $C_{\text{M}}$ by a constant phase element, which leads to improved fitting to real impedance measurements in practice. This modified single shell model has more recently been proposed by Ando et al. (2014) with the following impedance expression:


(9) $$\begin{array}{l} {\text{Z}}_{\text{single shell CPE}}(f)=\frac{R_{\text{E}}[1+{\left(2\pi f\right)}^{\alpha }Q\{(2R_{\text{I}}+R_{\text{E}})\cdot \cos(\frac{\pi }{2}\alpha )+{\left(2\pi f\right)}^{\alpha }QR_{\text{I}}(R_{\text{E}}+R_{\text{I}})\}]}{{\left\{{\left(2\pi f\right)}^{\alpha }Q(R_{\text{E}}+R_{\text{I}})\right\}}^{2}2{\left(2\pi f\right)}^{\alpha }Q(R_{\text{E}}+R_{\text{I}})\cdot \cos(\frac{\pi }{2}\alpha )+1}\\ \;-j\times \frac{{\left(2\pi f\right)}^{\alpha }QR_{E}^{2}\cdot \sin(\frac{\pi }{2}\alpha )}{{\left\{{\left(2\pi f\right)}^{\alpha }Q(R_{\text{E}}+R_{\text{I}})\right\}}^{2}2{\left(2\pi f\right)}^{\alpha }Q(R_{\text{E}}+R_{\text{I}})\cdot \cos(\frac{\pi }{2}\alpha )+1} \end{array}$$

A drawback of the use of CPEs in equivalent circuit models is that this hinders a convenient and straightforward interpretation. This issue is typically mitigated by converting the values of the CPE’s parameters to their corresponding apparent equivalent capacitance values using the following conversion:


(10) $$C_{\text{M},\text{apparant}}=Q{\left(2\pi f_{m}\right)}^{\left(\alpha -1\right)}\;,$$

where $f_{m}$ is the frequency that minimizes the imaginary part of the impedance. The value of $f_{m}$ is calculated as


(11) $$f_{m}=\frac{{\left(Q\left(R_{\text{E}}+R_{\text{I}}\right)\right)}^{\alpha }}{2\pi }\;.$$

Combining Eqs. (10) and (11), the apparent cell membrane capacitance is calculated as


(12) $$C_{\text{M},\text{apparant}}=Q^{\frac{1}{\alpha }}{\left(R_{\text{R}}+R_{\text{I}}\right)}^{\frac{1-\alpha }{\alpha }}\;.$$

Note that the underlying assumption of Eqs. (10) and (12) is that the frequency at which the imaginary part of the impedance is minimal remains the same after substitution of the CPE by $C_{\text{M},\text{apparant}}$ . This fractional single shell model with the calculation of the apparent capacitance has been used in many works since it was introduced by Ando et al. (Imaizumi et al., 2015; Ando et al., 2016; Meiqing et al., 2016; Watanabe et al., 2017; Watanabe et al., 2018; Li et al., 2019). The double shell model was proposed by Zhang and Wilson (1991) in an effort to take the electrochemical behavior of the vacuole into account. The impedance expression of the double shell model is reported in Inaba et al. (1995) and Wu et al. (2008). Despite being established over 30 years ago, the double shell model is still commonly used to analyze plant systems at present (Nouaze et al., 2022). Juansah et al. (2012) elaborated the double shell model further to include other fruit constituents. The authors report improved modeling of Garut fruit ripening with their proposed EEC. A fractional-order variant of this EEC has also been proposed (Cabrera-López and Velasco-Medina, 2019). Other elaborations of the double shell model, taking organelle resistances into account, have been proposed (Zhang et al., 1990; Harker and Maindonald, 1994), as well as a fractional variant of the double shell model substituting the capacitors with CPEs (AboBakr et al., 2017).

A bespoke choice should be made for the circuit to be used for analyzing a given set of plant EIS measurements, taking both the biophysical interpretation and quality of fit into consideration. That being said AboBakr et al. (2017) conducted a comparative evaluation of a selection of integer and fractional order EECs (including some of those discussed above) on electrochemical impedance measurements of several fruits. The authors concluded that the fractional single and double Cole models resulted in the best fit for the considered examples.

Electrode polarization effects at the interface between the plant and the measurement equipment are known to interfere with biological EIS measurements at low frequencies (Kuang and Nelson, 1998). Besides adjustments to the measurement setup, these effects are sometimes dealt with at the modeling stage through a serial addition in the EEC. Some novel EEC models with such additions were recently proposed by Ibba et al. (2020) and Sugiyama and Okajima (2022). Ibba et al.’s EEC model in their apple and banana ripening study (Ibba et al., 2020) consists of a Warburg element, modeling the interface between the fruit surface and the electrode, serially connected to a simple Voigt circuit. Sugiyama and Okajima (2022) proposed a fractional model that consists of a simple Voigt circuit, modeling the electrode–leaf contact, and a serially coupled fractionalized Hayden model describing the plant tissues. In later work, the same authors (Okajima and Sugiyama, 2023) simplified the part describing the plant tissues to a fractional single shell model, while adding a Warburg element in the part describing the electrode–leaf interface.

### 3.2 Parameter identification for plant EECs

A comprehensive equivalent-circuit-based analysis of EIS measurements relies on the adequate identification of the circuit parameters. The earliest described circuit parameterization methods were based on graphical measurements of the Nyquist plots (Cole, 1940). At present, the most common parameter estimation method is Complex Non-Linear Least Squares (CNLS) fitting, where the squared error between the measured impedance measurements and the simulated impedance spectra using the EEC and its parameters is minimized (Macdonald and Garber, 1977), i.e.:


(13) $$F\left(\kappa \right)=\sum_{i=0}^{N}|\text{Δ}Z_{i}{|}^{2}=\sum_{i=0}^{N}{\left(Z_{i,e}^{\text{Re}}-Z_{i,m}^{\text{Re}}\left(\kappa \right)\right)}^{2}+{\left(Z_{i,e}^{\text{Im}}-Z_{i,m}^{\text{Im}}\left(\kappa \right)\right)}^{2}$$

Here, $\text{Δ}Z_{i}$ is the difference between the experimental measurement and the equivalent circuit model’s corresponding impedance value for the i -th measured frequency. $Z_{i,e}^{\text{Re}}$ and $Z_{i,e}^{\text{Im}}$ are the real and imaginary parts of the experimental measurements at the i -th frequency, and $Z_{i,m}^{Re}\left(\kappa \right)$ and $Z_{i,m}^{Im}\left(\kappa \right)$ are the corresponding impedance values simulated by the circuit model using the parameter vector κ . The optimal EEC parameters for the considered circuit are argmin_κ_F(κ). The Levenberg-Marquart algorithm (Marquardt, 1963) is commonly used to solve this optimization problem, although many alternative algorithms have been proposed for bioimpedance analysis (Yousri et al., 2019; Gadallah et al., 2022; Ghoneim et al., 2022). Most plant EIS studies use the above method for circuit parameter identification, typically through commercial or freely available software, such as LEVM/LEVMW (MacDonald, 2013), EIS Spectrum Analyzer (Bondarenko and Ragoisha, 2005), LabView (Kodosky, 2020), or ZView (Johnson, 2000). A drawback of this method is that carefully chosen initial values for the equivalent electrical circuit model must be provided to the algorithm to ensure proper convergence. This parameter initialization can be done using graphical estimates, as in Ozier-Lafontaine and Bajazet (2005). Some researchers have circumvented the need for a good initialization by using optimization procedures that are less likely to get stuck in a local optimum due to an inadequate initial guess, such as evolutionary algorithms. The CNLS objective function in Eq. (13) is ubiquitously used and usually yields adequate results. However, it has also been subject to due criticism. In particular, an appropriate weighting of the two terms by the variance of the measurements (if available) or by the magnitudes of the experimental or model impedances (which are assumed to be proportional to the measurement variance) has been reported to be more appropriate in some works (Zoltowski, 1984; Orazem et al., 1994; Van Haeverbeke et al., 2021). This is especially the case when the measurements differ by several orders of magnitude, where a simple unit weighing causes F(κ) to be dominated by the larger impedance measurements.

In agricultural applications of EIS, it is desirable to conduct quick and efficient EIS measurements with inexpensive and portable hardware. EEC parameter estimation on incomplete measurements reduces the computational and hardware burden for such real-time and in-field applications. Freeborn et al. (2013) applied a non-linear least squares fitting method to accurately extract the parameters of the Cole model from the electrical current-excited step responses without requiring direct impedance measurements. Maundy et al. (2015) showed that the same could be done using only the impedance magnitudes, which also decreases the computational burden. Recently, Vastarouchas et al. (2019) developed a method to extract the Cole model parameters using only two measurements. Note that these methods are developed specifically for the Cole models described in Section 3.1 and are not applicable for general circuit parameter identification.

## 4 Statistical and machine learning methods for plant EIS

Researchers routinely apply various statistical methods when analyzing plant EIS measurements. These methods are typically applied to EIS features such as equivalent circuit parameters or impedance values at specific frequencies. They include various kinds of correlation analysis (Tomkiewicz and Piskier, 2012; Imaizumi et al., 2015; Hamed et al., 2016), as well as the comparison of multiple treatments using Analysis of Variance (ANOVA) (Jamaludin et al., 2014; Roy et al., 2019) with a range of posthoc tests (Harker and Forbes, 1997; Vozáry et al., 2007; Imaizumi et al., 2015; Meiqing et al., 2017).

Machine learning is the sub-discipline of artificial intelligence that focuses on automatically detecting patterns in data. In unsupervised learning, there is no outcome variable to be predicted. Here, patterns are detected within the unlabeled input data. Two important applications of unsupervised learning are clustering and dimensionality reduction. While being originally a classical statistical method, Principal Component Analysis (PCA) is an unsupervised learning algorithm commonly used to analyze plant EIS measurements (Cavalieri and Bertemes-Filho, 2021), where it aids in visualizing the impedimetric behavior resulting from different treatments in a study (Conesa et al., 2016; Hamed et al., 2016; Meiqing et al., 2016; Serrano-Pallicer et al., 2018; Ibba et al., 2020; Aparisi et al., 2021). It is a dimensionality reduction method that reduces the number of features in such a way that the most important information is retained. Some authors have applied dimensionality reduction methods to pre-process impedance features before conducting a supervised learning analysis (Guo et al., 2015; Conesa et al., 2016; Yu et al., 2016; Islam et al., 2018; Ochandio Fernández et al., 2019; Li et al., 2022). Alternatively, various methods have been used to select the most useful features before the analysis (Repo et al., 2014; Meiqing et al., 2016; Liu and Guo, 2017; Khaled et al., 2022).

In supervised learning, the goal is to predict an outcome variable that is often impractical to measure directly using a list of input variables (features). Depending on the nature of the response variable, a distinction is made between (i) regression problems, for which a continuous output value is to be predicted, and (ii) classification problems, for which the output is constrained to a discrete set of classes. After training (calibration) of supervised machine learning models, their predictive performance can be evaluated. For this, an appropriate performance metric should be selected. The model accuracy is the most common performance metric for plant EIS classification problems, which is the proportion of correct predictions relative to the total number of predictions. For regression problems, some commonly reported performance metrics are the coefficient of determination ( $R^{2}$ ) and the root-mean-square error (RMSE). The coefficient of determination is calculated for a set of N observations and regression model outputs as


(14) $$R^{2}=1-\frac{\sum_{i=1}^{N}{\left(Y_{i}-\hat{Y_{i}}\right)}^{2}}{\sum_{i=1}^{N}{\left(Y_{i}-\bar{Y}\right)}^{2}}$$

This can be interpreted as the proportion of the variance in the dependent variable observations $Y_{i}$ that the regression model outputs $\hat{Y_{i}}$ can explain. $\bar{Y}$ is the mean of the observations $Y_{i}$ . The RMSE is expressed in the same unit as the response variable and is calculated as


(15) $$\text{RMSE}=\sqrt{\frac{\sum_{i=1}^{N}{\left(Y_{i}-\hat{Y_{i}}\right)}^{2}}{N}}\;.$$

Another important consideration is the generalization ability of the model. A model generalizes well if it has a good predictive performance on new observations not used during model calibration. Overly complex models will fit the training data well but do not necessarily generalize well. This phenomenon is called overfitting. An appropriate model evaluation or selection, considering the possibility of overfitting, requires training and testing of the model to take place using separate sets of observations. In K -fold cross-validation, the full dataset is split into K different parts. The model is then consecutively trained on the dataset, excluding each of these parts and concurrently evaluating the excluded parts. The final performance estimation is the average evaluated performance on the K subsets. Statistical tests are also used for model selection. An example is a sequential F -test which can be used to compare models of varying complexity and assess the significance of newly introduced model elements.

We searched the literature for machine learning applications in EIS applied to plants. **Table 3** presents the research works retrieved. The vast majority of these applications predict fruit properties, such as ripeness or quality characteristics. Over half of these entries are studies published within the last 3 years, indicating that supervised machine learning methods are rapidly gaining interest in plant impedance spectroscopy. The most commonly used classification methods are Artificial Neural Networks (ANN) (Goodfellow et al., 2016) and Linear Discriminant Analysis (LDA) (Bishop and Nasrabadi, 2006). The most commonly used regression methods are Partial Least Squares (PLS) regression (Wold et al., 2001) and Multivariate Linear Regression (MLR) (Bishop and Nasrabadi, 2006). The number of measurements in the collected studies typically varies from tens to hundreds. Overall, high prediction performances are reported. The impedance values at specific frequencies were the most commonly used features to train the reported models. In several of these studies, the impedance spectrum was not completely measured, but only a few different frequencies were considered.

**Table 3:** Supervised machine learning approaches in plant electrochemical impedance spectroscopy.

Description	Type	Algorithm	Metric	Performance	Size	Year	Ref
Apple mouldy core	Classification	SVM	Accuracy	0.94	98	2016	(Yu et al., 2016)
Avocado ripeness	Classification	SVM	Accuracy	0.90	100	2018	(Islam et al., 2018)
Grapefruit freeze damage	Classification	MLP	Accuracy	1.00	180	2022	(Romero Fogué et al., 2022)
Lemon freeze damage	Classification	MLP	Accuracy	1.00	10	2019	(Ochandio Fernández et al., 2019)
Oil palm basal stem rot	Classification	LDA	Accuracy	0.86	240	2022	(Khaled et al., 2022)
Olive variety	Classification	MLP	Accuracy	1.00	90	2020	(Luna et al., 2020)
Orange freeze damage	Classification	MLP	Accuracy	1.00	270	2018	(Serrano-Pallicer et al., 2018)
Plant tissue discrimination	Classification	MLP	Accuracy	1.00	100	2020	(Cavalieri and Bertemes-Filho, 2020)
Rice seed vigor	Classification	LDA	Accuracy	0.90	100	2021	(Feng et al., 2021)
Strawberry ripeness	Classification	MLP	F1	0.72	923	2021	(Ibba et al., 2021)
Strawberry ripeness	Classification	MLR	Accuracy	0.773	150	2017	(González-Araiza et al., 2017)
Tangerine freeze damage	Classification	MLP	Accuracy	1.00	270	2021	(Aparisi et al., 2021)
Tomato ripeness	Classification	LDA	Accuracy	0.88	240	2019	(Li et al., 2019)
Wood chips	Classification	KNN	Accuracy	0.91	NA	2020	(Tiitta et al., 2020)
Apple moisture content	Regression	PLS	*R* ^2^	0.88	140	2018	(Reyes et al., 2018)
Apple soluble solids content	Regression	ELM	*R* ^2^	0.908	160	2015	(Guo et al., 2015)
Banana soluble solids content	Regression	LR	*R* ^2^	0.716	90	2014	(Jamaludin et al., 2014)
Crop leaf nitrogen content	Regression	MLR	*R* ^2^	0.94	111	2020	(Basak et al., 2020b)
Date acidity	Regression	MLP	*R* ^2^	0.938	800	2022	(Mohammed et al., 2022)
Durian dry matter content	Regression	PLS	RMSE	4.63%	120	2013	[Kuson and Terdwongworakul, 2013]
Korla pear hardness	Regression	NFS	*R* ^2^	0.911	61	2022	(Yu et al., 2022)
Korla pear soluble solids content	Regression	GRNN	*R* ^2^	0.974	300	2020	(Lan et al., 2020)
Leaf moisture content	Regression	MLR	*R* ^2^	0.959	28	2021	(Hao et al., 2021)
Lettuce Chlorophyll content	Regression	MLR	RMSE	1.05 *μ*g/L	70	2021	(Chowdhury et al., 2021)
Lime moisture content	Regression	PLS	*R* ^2^	0.934	82	2016	(Huong and Teerachaichayut, 2016)
Melon sugar content	Regression	ELM	*R* ^2^	0.887	480	2021	(Liu et al., 2021b)
Palm fruitlet oil content	Regression	LR	RMSE	5.71%	90	2022	(Chin-Hashim et al., 2022)
Peach firmness	Regression	CART	RMSE	1.59 N	200	2022	(Ivanovski et al., 2022)
Peach firmness	Regression	LR	MSE	0.67	200	2020	(Ivanovski et al., 2020)
Persimmon soluble solids content	Regression	LS-SVM	RMSE	0.97°Brix	105	2017	(Liu and Guo, 2017)
Pineapple sugars content	Regression	MLP	*R* ^2^	0.973	54	2016	(Conesa et al., 2016)
Sea buckthorn soluble solids	Regression	MLR	*R* ^2^	0.648	NA	2022	(Li et al., 2022)
Sweet potato moisture content	Regression	PLS	*R* ^2^	0.44	80	2018	(Reyes et al., 2018)
Tomato leaf nitrogen content	Regression	MLR	*R* ^2^	0.8374	35	2017	(Meiqing et al., 2017)
Tomato leaf phosphor content	Regression	MLR	*R* ^2^	0.864	34	2016	(Meiqing et al., 2016)
Tomato leaf potassium content	Regression	MLR	*R* ^2^	0.8561	34	2016	(Jinyang et al., 2016)

The encountered algorithms are K -nearest neighbors (KNN), (least-squares-) Support Vector Machine ((LS-)SVM), Linear Discriminant Analysis (LDA), Classification And Regression Trees (CART), Extreme Learning Machines (ELM), Neuro-Fuzzy System (NFS), Generalized regression neural network (GRNN), Partial Least Squares (PLS), Multi-layer Perceptron (MLP), and (Multivariate) Linear Regression (M)LR. When multiple classification problems or algorithms were considered, a single one was selected and reported per reference. If multiple algorithms were used, only the highest-performing one was reported. Data prepossessing steps are not reported.

## 5 Discussion

Valid EIS measurements of electrochemical systems conform to stability, causal, and time-invariance standards (Van Haeverbeke et al., 2022). Good practice dictates that the validity of measurements is verified before further analysis. EIS data validation through the Kramers–Kronig relations (Kronig, 1926) is an important standard in other EIS application areas (e.g., battery science). These relations evaluate the feasibility of computing the real part of the EIS measurements from the imaginary part and vice versa, which is a theoretical prerequisite for stable electrochemical systems. This data validation is rarely done for plant or other biological applications. The partial measurement of the impedance spectra (e.g., only measuring the real or imaginary parts to reduce the computational burden) described in Section 3.2 rules out the possibility of an adequate validation using the Kramers–Kronig relations.

The EIS field generally suffers from a lack of publicly available data. A notable exception, in the field of animal tissues, is the database of body tissue measurements compiled by Gabriel and Gabriel (1996). The further development of novel methods to analyze impedance spectroscopy measurements for plant applications would greatly benefit from publishing such collected data in well-maintained public databases accessible to other researchers. This could be done in a fashion similar to the MassIVE public database for mass-spectrometry measurements (Choi et al., 2020) or the many databases available for the bio-informatics community, such as Uniprot (Apweiler et al., 2004) and Genbank (Benson et al., 2012), to name a few.

Equivalent electrical circuits are still the standard tools for EIS analysis. Commonly applied EEC models in recent years are the double shell model and some fractionalized models such as the modified single shell model, the Cole model and distributed circuit element models with ZARC elements. The original integer-order Hayden and single-shell models are no longer commonly used. In some cases, arbitrarily complex EEC configurations are proposed in order to achieve an adequate fit to the plant EIS measurements (Islam et al., 2019). This results in the loss of the biophysical interpretation of the models, with a loss of the advantage of using EEC models over other non-linear models. Equation (10) is the widely applied mathematical formula proposed by Hsu and Mansfield (2001) for estimating the effective capacitance from CPE parameters in the fractional single shell model. Hirschorn et al. (2010) conducted a comparative evaluation of this formula and an alternative formula formerly proposed by Brug et al. (1984). A biological material was included in this study (human skin), where the formula by Brug et al. turned out to yield more satisfactory results. These results advocate further study on the most appropriate effective capacitance estimation procedure for plant systems.

A recent development in bio-impedance spectroscopy analysis is the use of the Distribution of Relaxation Times (DRT), which does not require the selection of a specific EEC model. Its strengths include the increased resolution in distinguishing different polarization processes and its general applicability. This analysis method has proven to be very effective in characterizing electrochemical power sources (Weiß et al., 2017). The initial development of a distribution of relaxation times analysis dates back to the beginning of the 19th century in work by von Schweidler (1907). The theory and methods were then further developed by the Cole brothers (Cole and Cole, 1941), among others. Schwan considered the theoretical description of a DRT for the analysis of biological tissues later in the last century (Schwan, 1957; Foster and Schwan, 1989). The improvement of EIS measurement technology and the development of adequate DRT deconvolution methods in the last decade have permitted its practical use. Recently, some promising evaluations of this method have been done for biological applications, such as the analysis of microbial fuel cells (Wang et al., 2022), animal tissues (Shi and Kolb, 2020), and cells in suspension (Ramírez-Chavarría et al., 2020). While this method has not yet been evaluated for plant EIS, recent developments in other fields suggest it could potentially become a valuable plant characterization method and provide informative features for machine learning models.

Due to the high dimensionality of EIS measurements and the typically limited (i.e., up to a few hundred) number of collected observations, current machine learning strategies benefit greatly from dimensionality reduction data preprocessing steps. Besides dimensionality reduction, there has not been much consideration for useful feature engineering strategies. Equivalent electrical circuit parameters, which contain the information from the impedance spectra after being fit, and distribution time constants are two interesting feature engineering strategies that can be evaluated in future work. A general remark on the machine learning approaches where classification is performed for ordinal outcomes, such as fruit ripeness or heartwood content of Scotts pine and the health state of oil palms (Khaled et al., 2018), is that the authors did not take the ordinality of the outcome variables into account (Frank and Hall, 2001). An exemplary consequence of neglecting this ordinality is that the misclassification of an overripe avocado as a firm avocado is not considered to be a larger error than the misclassification of a ripe avocado as an overripe avocado. Ordinality should be considered during the model development and evaluation stages (Cardoso and Sousa, 2011).

A few other criticisms of the works presented in **Table 3** are that i) sometimes no validation of the calibrated models on external datasets was performed (Basak et al., 2020b), ii) the necessary unit of the response variable when reporting the RMSE is often omitted, and iii) the results of some of the classification accuracies should be taken with a grain of salt, as the artificial classification settings may not be representative of actual practice. An example is the evaluation of freeze injury in citrus fruits. If the authors subject the fruits to intense freeze treatments, the differences between the impedance spectra of the two classes (damaged and non-damaged) are very large, such that even a simple model could achieve high classification performance. In this case, it is uncertain how the model would perform when faced with observations where the fruits are subjected to a lesser extent of freeze treatment.

Artificial neural networks are often reported in the new machine-learning-based impedimetric fruit quality monitoring trend. These appear to always be fully connected multi-layer perceptrons. Given the time-series nature of the EIS signals, other architectures, such as convolutional neural networks (CNN) or recurrent neural networks (RNN), may be more effective, as they have demonstrated high predictive performance in similar signal processing problem settings (Kiranyaz et al., 2019; Zhang et al., 2019).

Few studies have evaluated which non-destructive plant characterization methods complement each other well, bearing in mind the trade-off between increased depth and performance of a combination of complementary methods on the one hand and the increased labor and costs on the other hand (Srivastava and Sadistap, 2022). The rapid development and miniaturization of sensors and equipment hold promise for *in situ* applications and online *operando* plant monitoring. IoT data from different measured environmental variables (e.g., images, temperature, light intensity, and humidity) can be combined with electrochemical properties for agricultural management decision making, focusing on uncertainty quantification and interpretability in a bespoke probabilistic model in “smart farms” and greenhouses. As such, we can obtain highly accurate plant variables and quantifiable uncertainty, allowing for informed farm management decisions. To this end, we can take inspiration from Li-ion battery state of health research, where the authors probabilistically determined the state of health of the battery using impedance parameters in addition to the temperature and the state of charge of the battery (Zhang et al., 2022). If such models prove robust and reliable, it will lead to further advances in automation in “smart farms”.

## 6 Conclusion and future perspectives

A great deal of information on the physiological status of plants is contained in their electrochemical impedance spectra. One of the main challenges for plant EIS practitioners is extracting this information. In this work, we first provided an overview of the various physicochemical properties of plants that can be interrogated by EIS measurement on various plant organs. We then provided an overview of plant equivalent electrical circuit analysis as well as statistical and more recent machine learning approaches.

This paper proposed several suggestions to transfer knowledge and progress from the field of electrochemical power sources, which constitutes the most active area in EIS modeling research, to plant applications. These include adopting validation strategies, the fractionalization of equivalent circuit models, and the novel DRT method.

## Author contributions

MVH: conceptualization, writing – original draft. BDB: conceptualization, writing – editing, supervision. MS: conceptualization, writing – editing, supervision. All authors contributed to the article and approved the submitted version.

## Footnotes

1 Here, we consider potentiostatic EIS, while in galvanostatic EIS the input is an alternating current.
